# Genetic Diversity of Peach Cultivars from the Collection of the Nikita Botanical Garden Based on SSR Markers

**DOI:** 10.3390/plants10122609

**Published:** 2021-11-28

**Authors:** Aya A. Trifonova, Ksenia V. Boris, Natalia V. Mesyats, Valentina A. Tsiupka, Anatoly V. Smykov, Irina V. Mitrofanova

**Affiliations:** 1 Kurchatov Genomic Centre—NBG-NSC, 298648 Yalta, Russia; docboris@mail.ru (K.V.B.); valentina.brailko@yandex.ru (V.A.T.); irimitrofanova@yandex.ru (I.V.M.); 2 Vavilov Institute of General Genetics Russian Academy of Sciences, 119333 Moscow, Russia; 3 FSFIS “The Labor Red Banner Order Nikita Botanical Gardens—National Scientific Center of the RAS”, 298648 Yalta, Russia; vlasova_natali.zxcv@mail.ru (N.V.M.); selectfruit@yandex.ru (A.V.S.)

**Keywords:** peach, genetic resources, microsatellite markers, variability

## Abstract

The Nikita Botanical Garden (NBG) has a unique *Prunus* L. collection (peach, apricot, plum, cherry) comprising more than 3000 accessions. NBG is also a breeding center for stone fruits, including peach (*Prunus persica* (L.) Batsch). In the present study a set of 85 peach cultivars bred in NBG, Europe, and North America was analyzed using 12 SSR markers to assess their genetic diversity and relatedness. The detected polymorphism level was comparable to the previous estimates of genetic variability in peach cultivars. The average number of alleles per locus was 5.67, PIC value averaged 0.49, expected, and observed heterozygosity averaged 0.52 and 0.31, respectively. Among the detected alleles, 19 (27.94%) were rare and 12 (17.65%) were unique. All studied accessions except two could be identified with the used marker set. Cluster analysis revealed some groups according to the cultivars’ pedigrees. No clear differentiation of the studied sample according to geographic origin or fruit characteristics of peach cultivars was revealed. The results provide valuable information for identification and rational management of the material preserved in the NBG peach collection.

## 1. Introduction

The Nikita Botanical Garden (NBG) located on the southern coast of Crimea has a large gene pool collection of wild growing and cultivated plants, collected over 200 years. The favorable climate and geographical position provided the creation and successful development of a unique nurseries and acclimatization point for fruit crops in NBG on the border between Asia and Europe.

The NBG has unique *Prunus* L. collections (peach, nectarine, apricot, almond, plum, sweet and sour cherry), presented by more than 3000 species, hybrids, landraces, cultivars, and breeding forms of local and foreign breeding. The collection comprises 624 accessions of *Prunus persica* (L.) Batsch including cultivars and breeding forms of local breeding and cultivars from North America, Southern Europe, Central Asia, and the Caucasus. Additionally, more than 150 accessions of nectarine and ornamental peach are presented in the NBG collection.

The Nikita Botanical Garden is also a breeding center for stone fruits, including peach. Although in temperate climate peach growing is limited, breeding efforts are taken to improve peach cultivars for growing in unfavorable climatic conditions. There are 58 peach cultivars in the State Register for Selection Achievements Admitted for Usage in Russian Federation, of which 43 cultivars were bred by the researchers of the Nikita Botanical Garden. In this respect, conservation, maintenance, and study of the plant material, preserved in the collection as a source of genetic diversity for breeding is of special importance.

Microsatellites or SSR markers (Simple Sequence Repeats) based on variability of tandemly repeated DNA sequences have proved to be highly efficient for genetic diversity studies and cultivar identification in different fruit trees such as apricot [1], apple [2], almond [3], sweet cherry [4], and many others. Due to their abundance in the genome, simple and relatively low cost detection, microsatellites are still widely used for genetic analysis. To date, more than 500 SSRs have been developed for peach and other *Prunus* species [5,6,7,8,9,10], they have provided a very useful and convenient tool for analyzing genetic diversity in peach. The large-scale study of peach genetic variation in 224 North American and European commercial varieties, old Spanish varieties and several founders from the early USA peach breeding programs used 50 SSRs evenly distributed on the peach reference map [11]. The results revealed relatively low level of genetic variation and showed that these markers can be used to individually identify most genotypes and classify the cultivars according to key commercial fruit characteristics such as peaches, nectarines, and non-melting flesh peaches [11]. Another study used 48 SSRs, distributed over the peach genome, to investigate the difference in genetic diversity, and linkage disequilibrium (LD) among more than 600 Chinese, North American, and European cultivars and demonstrated higher level of genetic diversity and relatively fast decay of LD in the Oriental peach germplasm [12]. Different sets of SSR markers were also used to determine the genetic diversity and population structure of the breeding peach germplasm in the USA [13,14,15] and Brazil [16], to evaluate the genetic variation and linkage disequilibrium in Chinese peach cultivars and landraces [17,18,19,20] and for peach and nectarine cultivars fingerprinting [21]. The molecular genetic studies of the Nikita Botanical Garden peach collection were fragmented and most of the peach cultivars bred in NBG have not been analyzed using SSR markers.

In this study, 12 SSR markers were used to genotype 85 peach accessions from the NBG collection to assess their genetic diversity and population structure. The results will allow conserving and managing the collection more efficiently, because currently, the peach collection is being re-laid, so its genotyping is especially important.

## 2. Results

### 2.1. SSR Polymorphism and Cultivars Identification

Clear results of fragment analysis were obtained for 85 studied accessions. All studied SSR loci were polymorphic. A total of 68 alleles were detected across the 12 loci. The number of alleles per locus varied from 2 (*UDP97-402* and *UDP98-405*) to 9 (*CPPCT-022*) and averaged 5.67 (Table 1). Among the detected alleles, 19 (27.94%) were rare and 12 (17.65%) were unique. Two unique alleles were detected for cultivars ‘Earlicrest’ and ‘Kievskij Samyj Rannij’. Each of the accessions ‘Zheltoplodnyj Rannij’, (‘Druzhba Narodov’ × ‘Babygold-5 γ 40′) 97–120, ‘Jerseyglo’, ‘Starking Delicious’, ‘Sun German’, ‘Zerdabi’, ‘Gavazuri’, and ‘Kodru’ had one unique allele.

The number of genotypes per markers averaged 10.08. Only two genotypes were identified for *UDP98-405*: two cultivars ‘Kievskij Samyj Rannij’ and ‘Zheltoplodnyj Rannij’ had one genotype and the remaining accessions had the other one. The largest number of genotypes (14) was identified using *UDP96-005*, *BPPCT025,* and *CPPCT-022* markers. Polymorphism information content (PIC) varied from 0.04 (*UDP98-405*) to 0.68 (*UDP98-022*, *CPPCT-022*) and averaged 0.49. The observed heterozygosity (H_o_) values were not higher than 0.53 and averaged 0.31. Average value of expected heterozygosity (H_e_) was 0.52 and varied from 0.05 (*UDP98-405*) to 0.73 (*UDP98-022*) (Table 1).

The 12 selected SSR markers allowed the identification of 84 different genotypes among the 85 studied accessions. All the studied accessions had a unique SSR profile, except ‘Clyde Wilson’ and ‘Topaz’ which had identical fingerprints.

Dice genetic similarity coefficient was calculated for each pair of studied accessions. The maximum similarity (1) was revealed for the pair of accessions which had the same set of alleles (‘Clyde Wilson’–‘Topaz’). For three pairs of accessions, Dice genetic similarity coefficient was also high (‘Gagarinskij’–‘Kosmonavt 2′ (0.97); ‘Bokser’–‘German Titov’ and ‘Krymskij Shedevr’–‘Redcap’ (0.96)). The lowest level of similarity (0.13) was found for two pairs of accessions (‘Sovetski’j–‘Zerdabi’ and ‘Bokser’–‘Zerdabi’).

### 2.2. Genetic Differentiation and Structure

**Figure 1:**
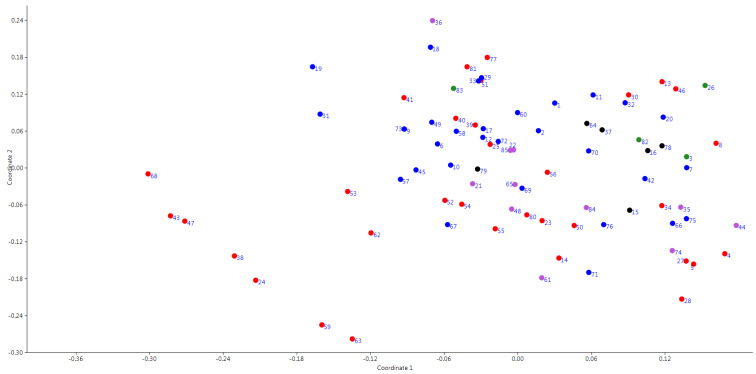
PCoA based on SSR data. Colors: red—the NBG accessions; blue—North American accessions; green—Caucasus accessions; violet—European accessions; black—hybrid forms and accessions of unknown origin. The numbers on the plot correspond to those in Table 3.

To elucidate genetic relationships among the studied peach cultivars the PCoA and cluster analyses were performed using the Dice similarity coefficient matrix. The result of the principal coordinate analysis is presented on Figure 1. On the PCoA plot, most of the studied accessions formed a general group that included peach cultivars of different origin. Except seven cultivars ‘Gagarinskij’, ‘Kosmonavt 2’, ‘Merkurij’, ‘Rekordist’, ‘Sovetskij’, ‘Steven Christian’ (NBG), and ‘Lyubimec Krasnodara’ (North Caucasian Federal Scientific Center of Horticulture, Viticulture, Wine-making (NCFSCHVW)) located separately on the plot.

**Figure 2:**
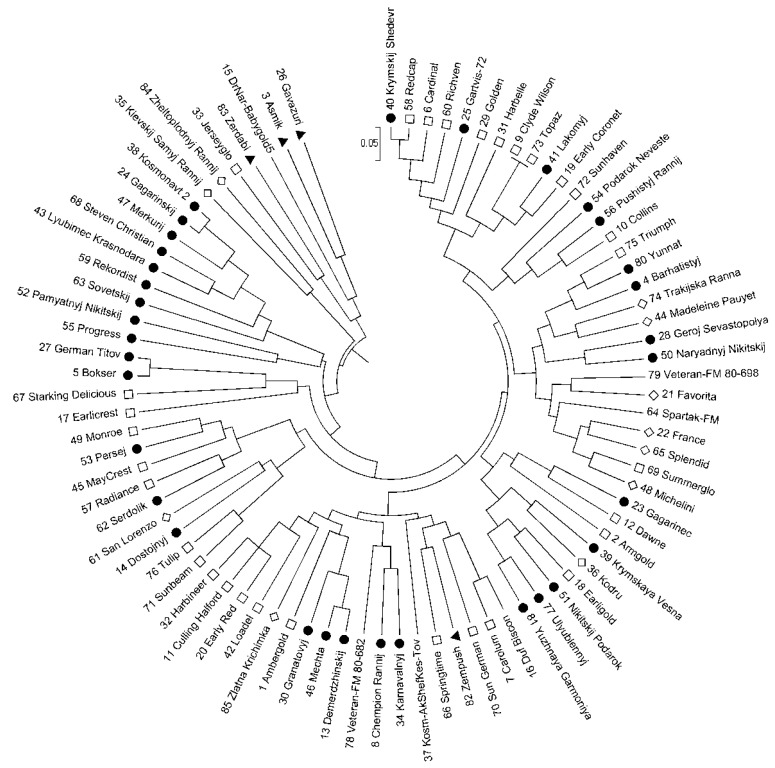
Dendrogram constructed according to SSR data by the UPGMA method. Symbols: ●—the NBG accessions; □—North American accessions; ▲—Caucasus accessions; ◊—European accessions; without symbols—hybrid forms and accessions of unknown origin. The numbers of accessions correspond to those in Table 3.

The UPGMA cluster analysis grouped samples in several clusters (Figure 2). The first one included five accessions: Caucasian landraces ‘Asmik’, ‘Zerdabi’, ‘Gavazuri’, hybrid form from NBG (‘Druzhba Narodov’ × ‘Babygold-5 γ 40′) 97–120, and cultivar ‘Jerseyglo’ (USA). Only two cultivars made up the second cluster: ‘Kievskij Samyj Rannij’ (Ukraine) and ‘Zheltoplodnyj Rannij’ (Moldavia). The third cluster included seven cultivars (‘Gagarinskij’, ‘Kosmonavt 2′, ‘Lyubimec Krasnodara’, ‘Merkurij’, ‘Rekordist’, ‘Sovetskij’, and ‘Steven Christian’) which were separated from the general group on the PCoA plot. Cultivars ‘Pamyatnyj Nikitskij’ and ‘Progress’ (NBG) were also included in the third cluster. The rest of the studied accessions form a cluster with a complex structure including cultivars of different origin, in which several subclusters based mainly on cultivars’ pedigrees can be distinguished.

**Figure 3:**
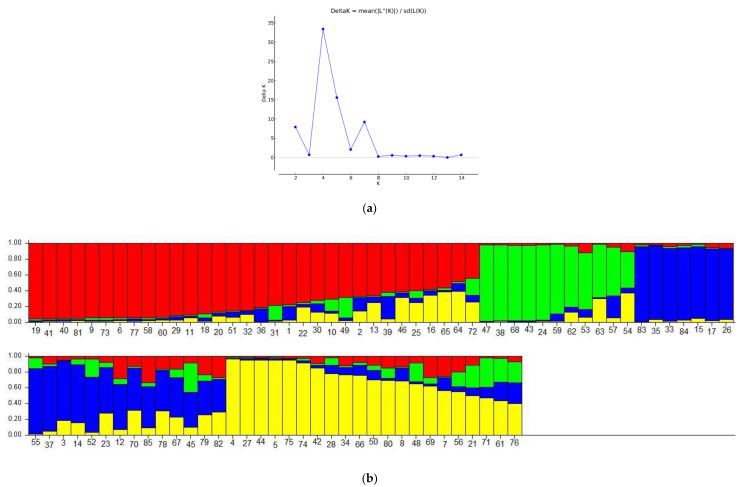
(**a**) Estimation of optimal number of clusters with the *deltaK* method. (**b**) Probability of cultivar assignment to one of the groups. The vertical axis represents the a posteriori probability of accessions membership in a group, the horizontal axis represents the studied accessions. The numbers of accessions correspond to those in Table 3.

The Bayesian clustering approach was applied to determine the genetic structure of the studied sample. SSR data analysis using the *deltaK* method demonstrated that the studied accessions most likely can be divided into four groups (Figure 3a). The first group (green bars) included eight cultivars ‘Gagarinskij’, ‘Kosmonavt 2′, ‘Lyubimec Krasnodara’, ‘Merkurij’, ‘Rekordist’, ‘Steven Christian’, separated from other accessions on the PCoA graph and on the dendrogram and ‘Persej’ and ‘Serdolik’ (NBG) (Figure 3b). Accessions of different origin ‘Gavazuri’, ‘Zerdabi’, (‘Druzhba Narodov’ × ‘Babygold-5 γ 40′) 97–120, ‘Jerseyglo’, ‘Kievskij Samyj Rannij’, ‘Zheltoplodnyj Rannij’, ‘Earlicrest’, ‘(Kosmicheskij × Ak Sheftalyu Kesma 84–107) × Tovarishch 92–2210′, and ‘Progress’ formed the second group (blue bars). The composition of this group was similar to the composition of the first and the second clusters on the dendrogram. The third group (yellow bars) consisted of seven cultivars: ‘Bokser’, ‘Barhatistyj’, ‘German Titov’, ‘Madeleine Pauyet’, ‘Trakijska Ranna’, ‘Triumph’, and ‘Loadel’. The last group (red bars) consists of 18 cultivars from NBG and North America. Other accessions included the components of several groups (Figure 3b).

A possible differentiation linked to geographic origin was investigated applying AMOVA among two groups: (1) North American cultivars and (2) NBG cultivars. The AMOVA results showed that only 4% of the total variation occurred between these groups (Table 2). Differentiation between groups with different fruit characteristics, (1) clingstone–non-melting, (2) clingstone–melting, (3) semifreestone–melting, and (4) freestone–melting, explained even less (1.6%) portion of the total variation (Table 2).

F_ST_ value between group of North American cultivars and group of NBG cultivars was 0.025. Pairwise F_ST_ values for groups with different fruit characteristics varied from 0.025 (between clingstone–non-melting and clingstone–melting groups) to 0.05 (between clingstone–non-melting and freestone–melting groups).

## 3. Discussion

The studied sample of 85 peach accessions represented mainly by the cultivars from the Nikita Botanical Garden (32) and North America (31) was rather diverse: 68 alleles were detected with 5.67 alleles per locus and H_o_ = 0.31.

These results are also consistent with previous studies of European and American peach germplasm, taking into account that nectarines and flat peaches were not included into our study. Microsatellites used for fingerprinting of 50 peach and nectarine cultivars detected 4.5 alleles per locus with the average heterozygosity value of 0.47 [22]. The later studies of 212 peach and nectarine cultivars using a set of 16 SSR markers and the extended sample of 224 cultivars using 50 markers revealed 7.3 and 6.36 alleles per locus, respectively, and similar H_o_ (0.35 vs. 0.34) [11,23]. The study of 94 native Spanish and foreign peach cultivars using 15 SSRs revealed 6.73 alleles per locus with average observed heterozygosity 0.23 [24]. Four alleles per locus and the heterozygosity mean value 0.33 were detected in the study of 112 peach cultivars from public and private US breeding programs using 20 SSR markers [14]. While for the 168 peach and nectarine cultivars and advanced selections from the University of Florida, 6.41 alleles per locus were detected using 36 SSRs with an average H_o_ = 0.41 [13].

Being the center of origin and domestication of peach, China has more genetically diverse peach germplasm. The study of 104 peach landraces from six Chinese geographical regions using 53 SSR markers revealed 6.4 alleles per locus, with an average PIC value 0.533 and the average genetic diversity 0.567 [19]. In the study of more than 600 peach accessions including Oriental (China, Japan, and Korea) and Occidental (Europe and USA) peach cultivars, landraces and wild species using 48 SSR markers 12.25 alleles per locus were detected with the average observed heterozygosity of 0.47, and an average expected heterozygosity—0.60 [12]. Unfortunately, cultivars from China and Central Asia presented in the NBG collection were not included in our study but may be of interest for further research.

The set of 12 SSR markers used in this study allowed detecting 84 unique genotypes among 85 accessions. Cultivars ‘Topaz’ and ‘Clyde Wilson’ derived from cultivar ‘Loring’ had identical alleles in all studied SSR loci, that may be due to the insufficient resolving power of the chosen markers, or misidentification of closely related cultivars in the collection. Still, 97.6% of the studied sample can be successfully identified using a set of 12 markers, even cultivars with common pedigree. For example, cultivars ‘Barhatistyj’ and ‘German Titov’ (‘Rochester’ × ‘Gum Kling’) had differences in two SSR loci and cultivars ‘Demerdzhinskij’, ‘Granatovyj’, and ‘Mechta’ (‘Valiant’ × ‘Favorita Morettini’) differed in four SSR loci.

Marker *CPPCT-022* was the most polymorphic and allowed to detect nine alleles. This marker has also shown a high level of polymorphism in previous studies [11,23,24]. Nevertheless, markers *UDP97-402* and *UDP98-405* that detected only two alleles made it possible to differentiate some accessions. Marker *UDP97-402* differentiated cultivar ‘Persej’ and marker *UDP98-405* differentiated cultivars ‘Kievskij Samyj Rannij’ and ‘Zheltoplodnyj Rannij’.

On the whole, the informativeness of the selected marker set (average PIC value 0.49) was comparable to other studies that used larger marker sets. In the study of 195 peach genotypes from the breeding pools of the University of Florida using a set of 36 SSR markers, PIC value was practically the same as in our study and averaged 0.48 [13]. In another study of 112 cultivars from the US using 20 SSRs, the average PIC value (0.32) was lower [14]. As well as in the study of 94 Asian peach accessions using the set of 34 SSRs (mean PIC = 0.40) [18]. While in the study of 94 peach cultivars including Spanish native peach and foreign commercial cultivars using 15 SSRs, the informativeness of the selected markers was higher with the mean PIC value 0.55 [24]. The same as in the study of Brazilian peach germplasm (204 genotypes) using a set of 10 markers, the PIC value averaged 0.59 [16].

The analysis of the obtained results with different statistical approaches revealed the genetic differentiation of cultivars ‘Gagarinskij’, ‘Kosmonavt 2’, ‘Merkurij’, ‘Rekordist’, ‘Sovetskij’, ‘Steven Christian’ (NBG), and ‘Lyubimec Krasnodara’ (NCFSCHVW). This group of cultivars was separated from other studied accessions on the PCoA plot, formed separate clusters on the dendrogram and on the Structure graph (Figure 1, Figure 2 and Figure 3b). These cultivars had no unique and rare alleles but had unique genotypes which differentiated them from other cultivars in four SSR markers from 12 used in the study.

Another group, that formed separate clusters on the dendrogram and on the Structure graph, included Caucasian landraces ‘Asmik’, ‘Zerdabi’ and ‘Gavazuri’, hybrid form (‘Druzhba Narodov’ × ‘Babygold-5 γ 40’) 97–120, and cultivars ‘Jerseyglo’, ‘Kievskij Samyj Rannij’, and ‘Zheltoplodnyj Rannij’ (Figure 2 and Figure 3b). Most of these accessions had unique and rare alleles. For example, hybrid form (‘Druzhba Narodov’ × ‘Babygold-5 γ 40’) 97–120 had one unique and six rare alleles. Cultivars ‘Asmik’, ‘Gavazuri’, and ‘Zerdabi’ had rare alleles (3, 3, and 4, respectively) and ‘Gavazuri’ and ‘Zerdabi’ also had one unique allele each.

Still, most of the detected groups were formed based on the cultivars’ pedigrees. The NBG cultivars ‘Gagarinskij’, ‘Rekordist’, ‘Steven Christian’, and ‘Sovetskij’, from a separate group mentioned above have cultivar ‘Golden Jubilee’ in their pedigree. Cultivar ‘Merkurij’, obtained by mutagenesis from ‘Sovetskij’, belongs to the same group. All cultivars derived from ‘Valiant’ × ‘Favorita Morettini’ (‘Demerdzhinskij’, ‘Granatovyj’ and ‘Mechta’) clustered together. As well as three out of five cultivars obtained from crossing ‘Veteran × Cardinal’: ‘Yuzhnaya Garmoniya’, ‘Ulyublennyj’, and ‘Nikitskij Podarok’.

Thus, no clear differentiation of cultivars according to their geographical origin was revealed (Table 2). This is not surprising, since many NBG cultivars were created using North American and European material. Additionally, the rest of the studied sample represents mainly cultivars from the USA and Europe and a few landraces. Previously, a small but significant differentiation (5.41% of variation (*p* < 0.001)) of North American and European peach cultivars was demonstrated between groups of peach cultivars from different provinces of Spain and cultivars from the USA [24]. The genetic diversity of the modern North American and European peach cultivars is limited because of the narrow genetic base, used in peach breeding programs in the 20th century [23].

Additionally, no significant differentiation of the studied sample by fruit characteristics including flesh type was revealed. This may be due to the high proportion of melting cultivars analyzed in our study (76 melting and 9 non-melting) and a small set of SSR markers used. Still, the number of alleles per locus for non-melting peaches was 4.08, and for melting 5.42. Comparing four groups, the most differentiated (F_ST_ = 0.05) were clingstone-non-melting peaches, and freestone-melting peaches. In previous studies of large collections, a differentiation between melting peaches, nectarines, and non-melting peaches was reported, with the higher polymorphism level and lower heterozygosity detected in non-melting peaches [11,23,24].

The present study provides the first insight into genetic variation of peach germplasm conserved in the collection of the Nikita Botanical Garden. SSR genotyping data will provide valuable information for proper characterization and effective management of the plant material preserved in the collection and for peach breeding program of the NBG, including protection of breeder’s intellectual rights. The results will also become the basis for further extended research on genetic diversity and genotyping of the NBG *Prunus* collection.

## 4. Materials and Methods

### 4.1. Plant Material and DNA Extraction

The plant material for the study included 85 peach accessions from the collection of the Nikita Botanical Garden with different fruit characteristics [25] (Table 3). Total genomic DNA was extracted from fresh young leaves according to the cetyltrimethylammonium bromide (CTAB) protocol [26] with minor modifications. DNA samples extracted were quantified using a NanoDrop OneC (Thermo Scientific, WI, USA) spectrophotometer.

### 4.2. SSR Analysis

A set of 12 SSR markers (BPPCT006, BPPCT007, BPPCT025, UDP96-001, UDP96-003, UDP96-005, UDP97-402, UDP97-403, UDP98-022, UDP98-405, UDP98-406, and CPPCT-022) [5,6,7] was used for genotyping.

PCR reactions were performed in T100 Thermal Cycler (BioRad, Hercules, CA, USA) in a final volume of 15 µL containing 20 ng of genomic DNA, 0.2 mM each dNTP, 1.6 mM MgCl_2_, Reaction Buffer (16,6 mM (NH4)_2_SO_4_; 67 mM Tris-HCL (pH 8.8 at 25 °C); 0.01% Tween20), 0.3 µM forward and reverse primers, and 0.5 U of BioTaq DNA polymerase (Dialat Ltd., Moscow, Russia). Forward primers were labeled with four different fluorescent dyes (6FAM, R6G, TAMRA, and ROX). The PCR conditions were initial denaturation for 4 min at 94 °C, followed by 34 cycles of 94 °C for 30 s, 57 °C for 40 s (except for *CPPCT-022*—50 °C) and 72 °C for 40 s, and a final extension of 5 min at 72 °C. All microsatellites were amplified separately and combined in multiplexes after PCR products were checked on 1.5% agarose gels in 1X TBE buffer and visualized by staining with ethidium bromide to test for the presence of PCR products.

Fluorescently labeled PCR products were separated by capillary electrophoresis on ABI Prism 3130xl (Applied Biosystems, Waltham, MA, USA). Fragment sizes were determined using GeneMapper v4.0 software (Applied Biosystems, Waltham, MA, USA).

### 4.3. Data Analysis

The frequencies of observed microsatellite alleles were measured using the GENALEX 6.41 software [27]. The polymorphism information content (PIC) was calculated as:

$$PIC=1-\overset{l}{\underset{i=1}{\sum }}P_{i}^{2}-\overset{l-1}{\underset{i=1}{\sum }}\overset{l}{\underset{j=i+1}{\sum }}2P_{i}^{2}P_{j}^{2}$$

where *P_i_* and *P_j_* are the population frequency of the *i*th and *j*th allele [28] in MS Excel. Expected (H_e_) and observed (H_o_) heterozygosity values of each microsatellite and frequency of rare (less than 5% of the accessions) and unique (less than 1%) alleles were calculated using the GENALEX 6.41 software [27].

Dice coefficient measured in PAST 3.16 software [29] was used for genetic similarity estimation, and to visualize genetic relationships among the studied accessions by an UPGMA (unweighted pair group method with arithmetic mean) clustering method, using MEGA 7 [30]. PAST 3.16 software [29] was used to carry out principal coordinates analysis (PCoA).

Genetic structure analysis of the collection was performed using Structure v.2.3.4 software [31]. From 1 to 15 clusters (K) with 30 replicates for each K were tested. The number of possible clusters was found as the result of 200,000 iterations of Markov chain Monte Carlo, taking into account genetic admixture and correlated allele frequencies. The first 20,000 generations were eliminated (burn-in). The optimal number of clusters was determined as recommended by Evanno et al. [32] using the online program Structure Harvester [33].

Differentiation of accessions depending on geographic origin (North American and NBG (other countries were represented by a small number of accessions)) and fruit traits (clingstone–non-melting, clingstone–melting, semifreestone–melting, and freestone–melting), was investigated with Analysis of Molecular Variance (AMOVA) in the GENALEX 6.41 software [27]. The threshold for statistical significance was determined by running 999 permutations. Pairwise F_ST_ estimates for groups of accessions were calculated using GENALEX 6.41 software [27].

## Figures and Tables

**Table 1:** Variability parameters calculated for 12 SSR markers in 85 peach accessions.

Locus	Number of Alleles	Allele Size Range, (bp)	Rare Alleles	Unique Alleles	H_o_	H_e_	PIC	Number of Genotypes
*BPPCT006*	7	118–139	1	3	0.25	0.38	0.36	11
*BPPCT007*	6	127–151	2	1	0.50	0.69	0.63	13
*BPPCT025*	7	178–199	1	2	0.34	0.66	0.63	14
*UDP96-001*	5	119–137	1	0	0.17	0.52	0.49	10
*UDP96-003*	6	125–151	2	1	0.37	0.53	0.49	11
*UDP96-005*	7	154–175	4	0	0.45	0.72	0.67	14
*UDP97-402*	2	134–146	0	0	0.22	0.22	0.19	3
*UDP97-403*	4	148–154	1	0	0.24	0.66	0.60	7
*UDP98-022*	7	127–142	1	2	0.38	0.73	0.68	13
*UDP98-405*	2	105–109	1	0	0.0	0.05	0.04	2
*UDP98-406*	6	99–120	2	1	0.26	0.38	0.36	9
*CPPCT-022*	9	251–299	3	2	0.53	0.72	0.68	14
All	68	-	19	12	-	-	-	-
Average	5.67	-	1.58	1.00	0.31	0.52	0.49	10.08

**Table 2:** Analysis of molecular variance (AMOVA) based on the 12 SSR loci of 85 studied peach accessions among inferred groups.

Source of Variation	d.f.	Sum of Squares	Estimated Variability	Percentage of Variation	*p* (Rand ≥ Data)
Geographic origin based					
Among groups	1	18.797	0.319	4	0.01
Within groups	62	533.640	8.607	96	0.01
Total	63	552.438	8.926	100	0.01
Fruit traits based					
Among groups	3	37.172	0.200	2	0.03
Within groups	81	716.770	8.849	98	0.03
Total	84	753.941	9.049	100	0.03

**Table 3:** Peach accessions taken into analysis.

№	Accession	Country/ Breeder	Pedigree *	Fruit Traits **		
1	Ambergold	USA	Red Grand × Royal May	Y	M	C
2	Armgold	USA	Flamingo × Springtime	Y	M	C
3	Asmik	Armenia	Chuguri op	W	M	C
4	Barhatistyj	NBG	Rochester × Gum Kling	Y	M	C
5	Bokser	NBG	(Rochester × Toscan Kling) op	Y	M	C
6	Cardinal	USA	Halehaven sp	Y	M	C
7	Carolum	USA	–	Y	N	C
8	Chempion Rannij	NBG	Chempion op F_1_ 1271	W	M	C
9	Clyde Wilson	USA	Loring mut	Y	M	F
10	Collins	USA	Jerseyland × [Raritan Rose × (J.H.Hale × Goldfinch) op]	Y	M	C
11	Culling Halford	USA	–	Y	M	C
12	Dawne	USA	–	Y	M	C
13	Demerdzhinskij	NBG	Valiant × FavoritaMorettini	Y	M	C
14	Dostojnyj	NBG	Zlatogor × Uspar-1	Y	N	C
15	(Druzhba Narodov × Babygold-5 γ 40) 97–120	NBG	Druzhba Narodov × Babygold-5	W	N	C
16	Duf Biscon	–	–	C	M	F
17	Earlicrest	USA	Springcrest mut	Y	M	C
18	Earligold	USA	(Luken’s Honey × July Elberta) × [(Luken’s Honey × July Elberta) × Robin]	Y	M	C
19	Early Coronet	USA	Coronet mut	Y	M	C
20	Earlired	USA	Redhaven × [Halehaven × (Halehaven × Oriole)]	Y	M	C
21	Favorita	Italy	Giala di Firenze × Fertilia I	Y	M	C
22	France	France	–	W	M	F
23	Gagarinec	NBG	Pushistyj Rannij × Grinsboro	W	M	S
24	Gagarinskij	NBG	Golden Jubilee sp	Y	M	C
25	Gartvis-72	NBG	Nikitskij × Early Rivers	Y	M	S
26	Gavazuri	Georgia	–	Y	M	F
27	German Titov	NBG	Rochester × Gum Kling	Y	M	C
28	Geroj Sevastopolya	NBG	Rot Front × Pobeditel	W	M	S
29	Golden	USA	–	Y	M	C
30	Granatovyj	NBG	Valiant × Favorita Morettini	Y	M	C
31	Harbelle	Canada	Sunhaven sp	Y	M	S
32	Harbinger	Canada	Cherryred × (Jerseyland × Mayflower)	Y	M	C
33	Jerseyglo	USA	Jefferson × Loring	Y	M	F
34	Karnavalnyj	NBG	Veteran × Cardinal	Y	M	C
35	Kievskij Samyj Rannij	Ukraine	Kashchenko 208 × Gross Minion	W	M	C
36	Kodru	Moldavia	–	Y	M	C
37	(Kosmicheskij × Ak Sheftalyu Kesma 84-107) × Tovarishch 92–2210	NBG	(Kosmicheskij × Ak Sheftalyu Kesma) × Tovarishch	Y	M	C
38	Kosmonavt 2	NBG	Triumph × Arabka	Y	M	C
39	Krymskaya Vesna	NBG	Veteran sp	Y	M	C
40	Krymskij Shedevr	NBG	Mayflower op № 254 (in vitro)	Y	M	C
41	Lakomyj	NBG	Redhaven × Kudesnik	Y	M	C
42	Loadel	USA	Lovell op	Y	N	C
43	Lyubimec Krasnodara	Russia	Gayar-9 op	Y	M	C
44	Madeleine Pauyet	France	Mayflower mut	W	M	C
45	Maycrest	USA	Springcrest mut	Y	M	C
46	Mechta	NBG	Valiant × Favorita Morettini	Y	M	F
47	Merkurij	NBG	Sovetskiy mut	Y	M	F
48	Michelini	Italy	–	W	M	F
49	Monroe	USA	Rio Oso Gem × (Shippers Late Red × Sunhigh)	Y	M	F
50	Naryadnyj Nikitskij	NBG	Veteran × Cardinal	Y	M	C
51	Nikitskij Podarok	NBG	Veteran × Cardinal	Y	M	C
52	Pamyatnyj Nikitskij	NBG	Pamyat’ ob Ottse op	C	M	C
53	Persej	NBG	Rot Front op	Y	M	F
54	Podarok Neveste	NBG	Natusya op	W	M	C
55	Progress	NBG	Laureat op	W	N	C
56	Pushistyj Rannij	NBG	Rochester × almond-pers. hybrid pollen	W	M	C
57	Radiance	USA	Belle × Greensboro	W	M	S
58	Redcap	USA	Southland × Dixired	Y	N	C
59	Rekordist	NBG	Golden Jubilee sp	Y	M	S
60	Richven	USA	–	Y	M	C
61	San Lorenzo	Spain	–	W	M	S
62	Serdolik	NBG	Zlatogor × Uspar-1	Y	N	C
63	Sovetskij	NBG	Golden Jubilee × Narindzhi Pozdniy	Y	M	F
64	Spartak × Favorita Morettini 92-1281	NBG	Spartak × Favorita Morettini	Y	M	C
65	Splendid	Romania	J.H.Hale × Peen Too	W	M	F
66	Springtime	USA	(Luken’s Honey × July Elberta) × Robin	W	M	C
67	Starking Delicious	USA	July Elberta mut	Y	M	S
68	Steven Christian	NBG	Golden Jubilee sp	Y	M	S
69	Summerglo	USA	Collins × Red Slovenia	Y	M	F
70	Sun German	USA	–	Y	N	C
71	Sunbeam	USA	Slappey × Arp	Y	M	S
72	Sunhaven	USA	Redhaven × (J.H. Hale × Halehaven)	Y	M	S
73	Topaz	USA	Loring sp	Y	M	S
74	Trakijska Ranna	Bulgaria	–	W	M	S
75	Triumph	USA	Alexander op	Y	M	S
76	Tulip	USA	Sunbeam op	Y	M	C
77	Ulyublennyj	NBG	Veteran × Cardinal	Y	M	C
78	Veteran × Favorita Morettini 80–682	NBG	Veteran × Favorita Morettini	Y	M	C
79	Veteran × Favorita Morettini 80–698	NBG	Veteran × Favorita Morettini	Y	M	F
80	Yunnat	NBG	Rot Front × Triumph	Y	M	C
81	Yuzhnaya Garmoniya	NBG	Veteran × Cardinal	Y	M	C
82	Zempush	Azerbaijan	–	W	M	C
83	Zerdabi	Azerbaijan	–	Y	N	C
84	Zheltoplodnyj Rannij	Moldavia	–	Y	M	C
85	Zlatna Krichimka	Bulgaria	–	Y	M	S

* op—open pollination; sp—self pollination; mut—mutation. ** W—white flesh; Y—yellow flesh; C—cream flesh; M—melting flesh; N—non-melting flesh; F—freestone; S—semifree; C—clingstone.

## Data Availability

Raw data are available on request from the corresponding author.
